# Ethylene glycol nanofluids dispersed with monolayer graphene oxide nanosheet for high-performance subzero cold thermal energy storage†

**DOI:** 10.1039/d1ra04484b

**Published:** 2021-09-14

**Authors:** Jingyi Zhang, Benwei Fu, Chengyi Song, Wen Shang, Peng Tao, Tao Deng

**Affiliations:** State Key Laboratory of Metal Matrix Composites, School of Materials Science and Engineering, Shanghai Jiao Tong University Shanghai 200240 China taopeng@sjtu.edu.cn dengtao@sjtu.edu.cn

## Abstract

Ethylene glycol (EG) nanofluids have been intensively explored as one of the most promising solid–liquid phase change materials for subzero cold thermal energy storage (CTES). However, the prepared nanofluids usually suffer from a large supercooling degree, a long freezing period, reduced storage capacity and poor dispersion stability. Herein, we overcome these issues by developing stable EG nanofluids that are uniformly dispersed with low concentrations of monolayer ethanol-wetted graphene oxide nanosheets. The homogeneously dispersed monolayer sheet not only improves the thermal conductivity of the nanofluids (12.1%) but also provides the heterogeneous nucleation sites to trigger the crystal formation, thereby shortening the freezing time and reducing the supercooling degree. Compared with the base fluid, the nanofluids have reduced the supercooling degree by 87.2%, shortened the freezing time by 78.2% and maintained 98.5% of the latent heat. Moreover, the EG nanofluids have retained their initial stable homogeneous dispersion after repeated freezing/melting for 50 cycles, which ensures consistent CTES behavior during long-period operations. The facile preparation process, low loading requirement and consistent superior thermophysical properties would make the EG nanofluids loaded with monolayer graphene oxide sheets promising coolants for high-performance phase change-based CTES.

## Abstract 2

Homogeneously dispersed monolayer graphene oxide sheet in ethylene glycol solution enable a high-performance cold thermal energy storage.

## Graphical abstract

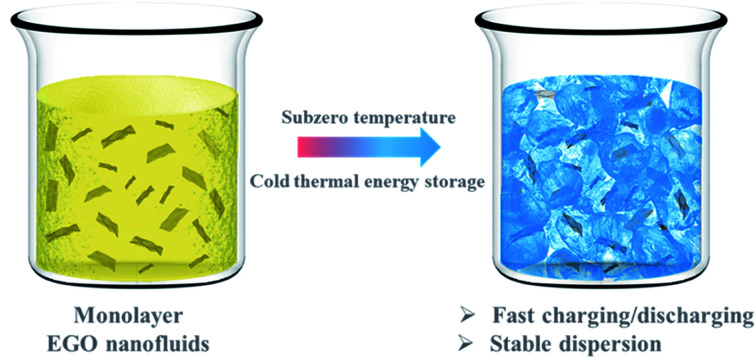

## Introduction

Cold thermal energy storage (CTES) has played a crucial role in a wide range of applications including waste heat recovery, food preservation and central air-conditioning.^1–3^ Solid–liquid phase change-based CTES has received tremendous research attention due to a large amount of latent heat released or absorbed during the phase transition and nearly constant heat-releasing temperature.^4–8^ In a typical charging process, the phase change materials (PCMs) are first placed within the low-temperature environment to be rapidly cooled down to a supercooled temperature (*T*_S_). Under such temperature, liquid PCM is supercooled to provide the driving force for the nucleation of solid PCM crystals. As the nucleation process proceeds, the temperature of PCM quickly returns to the phase change temperature (*T*_P_) and the solidification process occurs. During the liquid-to-solid phase transition, the temperature of PCM fluctuates near *T*_P_ within a narrow range. After full solidification, the temperature of the solid PCM further slowly reduces towards the ambient temperature. Besides the large heat storage capacity and a suitable heat-releasing temperature, high-performance CTES also requires that PCMs should have a small supercooling degree (Δ*T*), which is the difference between *T*_S_ and *T*_P_, thus, the solidification process can proceed with a reduced input of cold thermal energy.^9,10^ In addition, a large thermal conductivity is desired such that the released latent heat can be effectively exchanged with the ambient environment and the discharging period can be shortened.^11,12^

Among various PCMs investigated so far, ethylene glycol (EG) solutions are one of the most promising candidates for subzero CTES because of their appropriate phase change temperature, large latent heat capacity, chemical stability and abundance.^3,13,14^ In particular, the thermophysical properties of EG–water solutions, such as thermal conductivity and phase change temperature can be tuned by tailoring the chemical composition.^15^ It has been reported that the solution containing 25 vol% of EG and 75 vol% of water (25 vol% EG coolant) could combine high thermal conductivity of water and the low phase change temperature of EG, and achieve a subzero storage temperature of ∼−16 °C.^5^ Such EG solutions, however, still suffer from a large supercooling degree and low thermal conductivity. Dispersing functional nanoparticles into the base fluids to prepare nanofluids has been pursued to address these issues.^16–27^ For example, TiO_2_ and ZnO nanoparticles have been added to the EG–water solution to reduce the supercooling degree and improve thermal conductivity.^28,29^ Compared with zero-dimensional nanoparticles, two-dimensional graphene oxide (GO) nanosheets have shown superior heterogeneous nucleation effect and an enhancement in heat transfer,^30,31^ but they have a strong tendency to stack and aggregate due to the π–π interaction between adjacent layers.^32,33^ The aggregated sheets tend to precipitate under gravity leading to the loss of the expected improvement of CTES performance and even blockage of the CTES system.^34,35^ Usually, time-consuming surface modification with various surfactants is required to keep their dispersion stability.^36,37^ The surface ligands or polymer chains, however, would interfere with the liquid–solid phase change, delay the solidification process^38,39^ and impede heat transfer.^40^ Therefore, developing self-dispersible GO–EG nanofluids with combined low supercooling, high thermal conductivity, large latent heat and good dispersion stability is critical for high-performance solid–liquid phase change-based subzero CTES.

Herein, we report the preparation of EG nanofluids uniformly dispersed with monolayer ethanol-wetted GO (EGO) and demonstrate that such nanofluids simultaneously possess the desired features, such as a low supercooling degree, high thermal conductivity, large latent heat and good dispersion stability for high-performance subzero CTES. The monolayer EGO sheets not only provided numerous heterogeneous nucleation sites but also improved the thermal conductivity of nanofluids, thereby reducing the supercooling degree and shortening the freezing time. Compared with the base fluids, the supercooling degree and freezing time of the nanofluids loaded with 3.75 mg mL^−1^ were reduced by 87.2% and 78.2%, respectively, which are superior to the CTES performance of EG and water-based nanofluids reported so far. Moreover, the low loading requirement enables the nanofluids to retain large latent heat. In addition, the EGO nanofluids maintain stable uniform dispersion after repeated freezing–melting for 50 cycles. The stable dispersion benefits achieve consistent CTES performance of nanofluids and paves the way for their practical applications.

## Materials and methods

### Chemical materials

Nano-graphite (*D*_50_ < 400 nm) was purchased from Shanghai Macklin Biochemical Co., Ltd. Sodium nitrate (NaNO_3_), potassium permanganate (KMnO_4_), sulfuric acid (H_2_SO_4_, 95–98 wt%) and ethanol (≥99.5 wt%) were supplied by Sinopharm Chemical Reagent Co., Ltd. Ethylene glycol and hydrogen peroxide solution (30–31 wt%) were purchased from Aladdin Industrial Co., Ltd. Hydrochloric acid (36–38 wt%) was ordered from Yonghua Chemical Technology Co., Ltd. All reagents were used as received.

### Synthesis of EGO and WGO

GO was synthesized through chemical exfoliation of natural graphite powders by using a modified Hummer's method.^41^ The as-prepared GO was dipped into 10 vol% HCl aqueous solution for 12 h and the solution was then centrifuged at 8000 rpm for 10 min. The obtained precipitates were dispersed in anhydrous ethanol through mild sonication for 5 min followed by centrifugation at 8000 rpm for 15 min. Such washing and centrifugation processes were repeated for 5 times. The obtained precipitates were then dried in air at room temperature for 5 h. The obtained products were named as ethanol-wetted GO (EGO). The deionized water-wetted GO (WGO) was prepared by the same way except that deionized water instead of ethanol was used as the dispersing solvent during the washing processes.

### Preparation of nanofluids

In order to determine the optimum volume fraction of EG, the phase change temperatures of 15%, 25%, 50% and 75% EG solutions were measured (Fig. S1†). It was found that 25% EG solution had a suitable phase change temperature (−12.5 °C), but the freezing point of 15% EG solution was only −2.9 °C, which could not meet the cooling requirements in drug preservation and central air-conditioning applications.^2^ For 50% and 75% EG solutions, no phase change was observed within the tested temperature range from −50 °C to 20 °C. The amount of EG also affects other physical properties of the mixed coolants such as thermal conductivity, latent heat, and viscosity. A large volume fraction of EG would lead to decreased thermal conductivity, lower latent heat, and higher viscosity,^42^ which would limit the CTES performance and application of prepared coolants. After taking these factors into consideration, 25 vol% EG solution was selected as the base fluid.

To prepare the nanofluids, the obtained EGO was firstly dispersed within pure EG to form the EGO–EG dispersion with 5 different concentrations (3 mg mL^−1^, 6 mg mL^−1^, 9 mg mL^−1^, 12 mg mL^−1^, 15 mg mL^−1^). Water was subsequently added to the dispersion to yield a base fluid composition of EG (25 vol%)–water (75 vol%). The concentration of EGO in nanofluids was diluted to 0.75 mg mL^−1^, 1.50 mg mL^−1^, 2.25 mg mL^−1^, 3.0 mg mL^−1^ and 3.75 mg mL^−1^, respectively. The WGO nanofluids with five concentrations were also prepared.

### Characterization and property measurement

The microstructure of EGO and WGO was observed using a transmission electron microscope (TEM, JEM-2100F). An atomic force microscope (AFM, Dimension Icon & FastScan Bio) was used to measure the thickness of GO sheets. A hot disk method (Hot disk 2500S) was applied to measure the thermal conductivity of the prepared nanofluids. The measurement was performed at least three times for each sample. The zeta potential measurement of all samples was conducted on a zeta potential analyzer (Brookhaven, Omni, USA). The contact angle was measured using a high-speed camera (AOS Technologies AG, S-VIT LS) at ambient temperature and the obtained image was further analyzed by the CorelDRAW software (X6).

### Cold thermal energy storage performance measurement

Five identical glass tubes with a diameter of 18 mm and a height of 152 mm containing 3 mL of nanofluids with different concentrations were immersed in a low-temperature thermostat bath (BILON-W-504S) that was stabilized at −19 °C. T-type thermocouples that were connected with a multichannel data acquisition system (Agilent 34972A) were used to monitor the temperature change in real-time. The thermocouple was immersed inside the nanofluids and fixed in the middle of the glass tube. The key CTES performance indicators including supercooling degree, solidification time and freezing point were analyzed from the measured temperature evolution profiles. The phase change enthalpy was measured from 20 °C to −50 °C using a differential scanning calorimeter (DSC 204 F1) under a cooling rate of 5 °C min^−1^. The uncertainty associated with measuring the freezing time and supercooling degree is provided in Table S1.†

## Results and discussion

### Preparation and characterization of EGO nanofluids

**Fig. 1:**
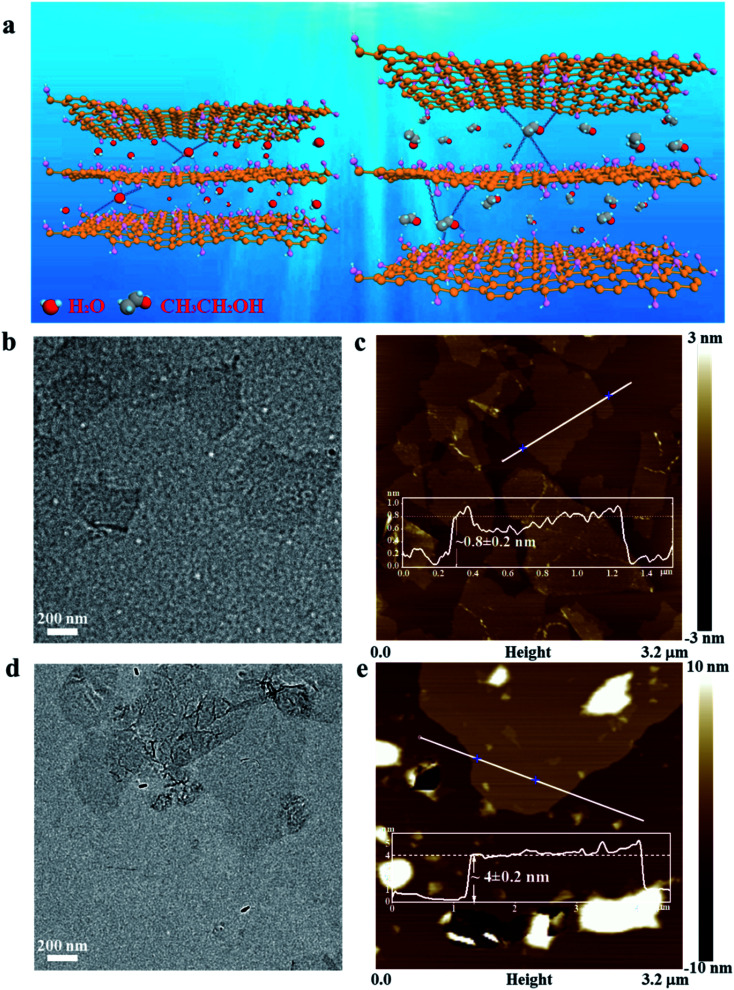
(a) Schematic showing the intercalation of water and ethanol molecules between GO sheets. (b) TEM image of monolayer EGO sheet. (c) AFM image and height profile of EGO sheets. (d) TEM image of stacked WGO sheets. (e) AFM image and height profile of WGO sheets.

EGO sheets were prepared by exfoliating graphite using a modified Hummer's method.^41^ Unlike conventional water-wetted GO (WGO) sheets that were prepared by washing and dispersing exfoliated GO sheets within deionized water, herein, ethanol was used as the washing and dispersing solvent. As shown schematically in Fig. 1a, the ethanol molecule has a larger molecular volume and weaker interaction with the oxygen-containing groups on the surface of GO sheets than with the water molecule, which helps enlarge the interlayer spacing of EGO. The increased layer distance helps weaken the van der Waals attraction between adjacent layers and facilitates exfoliating stacked layers into a monolayer GO sheet.^43,44^ TEM observations showed that EGO sheets were separated from each other and appeared nearly transparent (Fig. 1b). The AFM image in Fig. 1c shows that the EGO sheets have a height of 0.8 ± 0.2 nm, proving that the EGO sheets are indeed exfoliated into monolayers. By contrast, the WGO sheets were stacked together showing a much higher image contrast (Fig. 1d). The thickness of WGO sheets was measured to be 4.1 ± 0.2 nm (Fig. 1e), which is equivalent to 4–5 stacked layers. When the EGO sheets were homogeneously dispersed into the EG base fluid, a layer of EG molecules adsorbed around the surface of EGO sheets before being further diluted with water. The adsorbed EG layer helps to maintain the initial uniform dispersion state of the monolayer EGO sheet in the coolant.^45,46^ As shown in Fig. S2,† although both the as-prepared EGO and WGO sheets can be initially dispersed in the coolant without forming any visible agglomeration through ultrasonication, the color of WGO nanofluids is much darker than that of the EGO nanofluids. The dark color is related to the increased size of the conjugated π-domains for WGO sheets, which also strengthens the π–π interaction among GO layers and undermines the dispersion stability of conventional WGO nanofluids.^47^

### Subzero CTES performance

**Fig. 2:**
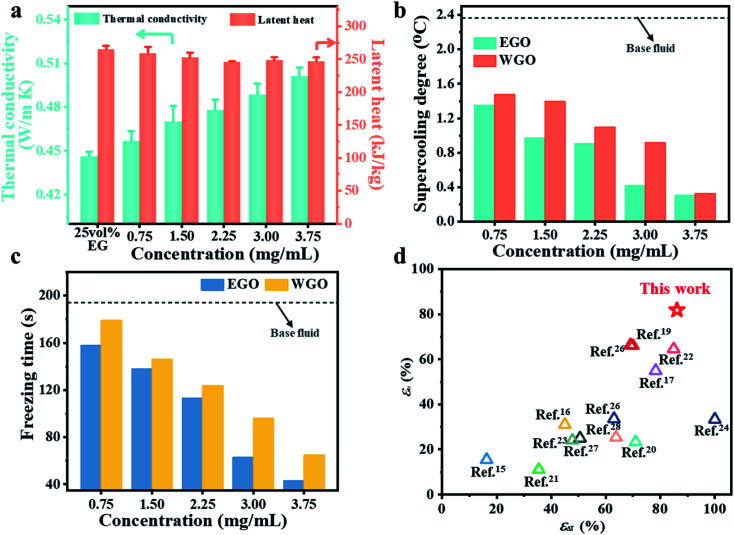
(a) Comparison of thermal conductivity and latent heat for EG solution and EGO nanofluids with various concentrations. (b) Supercooling degree of EGO and WGO nanofluids (grey dashed line: the supercooling degree of base fluid). (c) Freezing time of EGO and WGO nanofluids (grey dashed line: the freezing time of base fluid). (d) Comparison of reduction percentage of the supercooling degree (*ε*_Δ*T*_) and the freezing time (*ε*_tf_) between EGO nanofluids and other reported water and EG nanofluids.

High-performance CTES requires that PCMs should simultaneously possess a low supercooling degree to trigger the crystal growth, high thermal conductivity to reduce freezing time, and large latent heat to store cold thermal energy. Fig. 2a presents that the thermal conductivity of EGO nanofluids gradually rises with increasing concentration of EGO sheets. With a concentration of 3.75 mg mL^−1^, the effective thermal conductivity of nanofluids reached ∼0.5 W m^−1^ K^−1^, which is 12.1% larger than that of the base fluid. The solid–liquid phase change process was characterized by DSC measurement for both EGO and WGO nanofluids. As shown in Fig. S3,† EGO and WGO nanofluids show similar DSC curves and the phase change temperature gradually increases with increasing concentration of GO sheets. Fig. 2a shows that the effective latent heat of the nanofluids slightly decreases with the increasing concentration of EGO sheets. When the concentration was within the range of 2.25–3.75 mg mL^−1^, the latent heat was stabilized at around ∼250 kJ kg^−1^. Compared with the base coolant fluids, the negligible decrease of latent heat for the nanofluids should be attributed to the low concentration of EGO sheets. Similarly, EGO and WGO nanofluids retain nearly the same density and viscosity as that of the 25 vol% EG base fluid (Table S2†).

To investigate the CTES performance, glass tubes containing nanofluids were placed in a thermostatic bath that was stabilized at −19 °C, and the time-dependent temperature profiles of the nanofluids were recorded. Fig. S4† presents that all the tested samples were firstly cooled down to *T*_S_ to provide the driving force for the formation of the crystal nuclei. Subsequently, the temperature of nanofluids returned to the *T*_P_ and fluctuated near that temperature until the entire crystallization process was completed. All the samples showed nearly the same *T*_P_ around −16 °C and the number of added GO sheets has little influence. The freezing time and the supercooling degree decreased with increasing content of GO for both EGO and WGO nanofluids. Fig. 2b shows a comparison of the supercooling degree of EG nanofluids loaded with the same concentration of EGO and WGO sheets. It can be seen that both EGO and WGO sheets could serve as heterogeneous nucleation centers to reduce the supercooling degree. By comparison, EGO nanofluids have demonstrated a better reduction effect of the supercooling degree than WGO nanofluids under the same concentration of GO sheets. With a concentration of 3 mg mL^−1^, the supercooling degree of the nanofluids loaded with EGO sheets fell from 2.368 °C for the base fluid to 0.416 °C, and that of WGO nanofluids dropped to 0.916 °C.

Fig. 2c compares the freezing time (*t*_f_) for EGO and WGO nanofluids. A similar gradual reduction of the freezing time with increasing concentration of GO sheets was observed, and with the same concentration, EGO nanofluids have demonstrated a shorter freezing time than the WGO nanofluids. Under the same concentration of 3 mg mL^−1^, the freezing time for EGO and WGO nanofluids declined from 194 s for the base fluids to 65 s and 96 s, respectively. The contraction of *t*_f_ should be ascribed to the accelerated nucleation and crystal growth of solid PCMs during the solidification process. The dispersed monolayer EGO sheet not only provides more sites than the stacked WGO sheets for rapid heterogeneous nucleation of PCM crystals but also facilitates the formation of heat conduction paths to effectively transfer the released latent heat out to the ambient environment during the solidification process, thus promoting the fast growth of PCM crystals.

The uniformly dispersed monolayer EGO sheet offers the unique opportunity to simultaneously decrease the supercooling degree and shorten the freezing time at low concentrations while maintaining the large latent heat capacity of the PCM coolant. Fig. 2d shows that the EGO nanofluids (3.75 mg mL^−1^) have superior improvement of supercooling (*ε*_Δ*T*_, Note S1†) and freezing time (*ε*_tf_, Note S1†) than the other water and EG-based nanofluids reported so far. In particular, the homogenous dispersion of the monolayer EGO sheet helped significantly reduce the supercooling degree of the PCM coolant.

### Stability performance

**Fig. 3:**
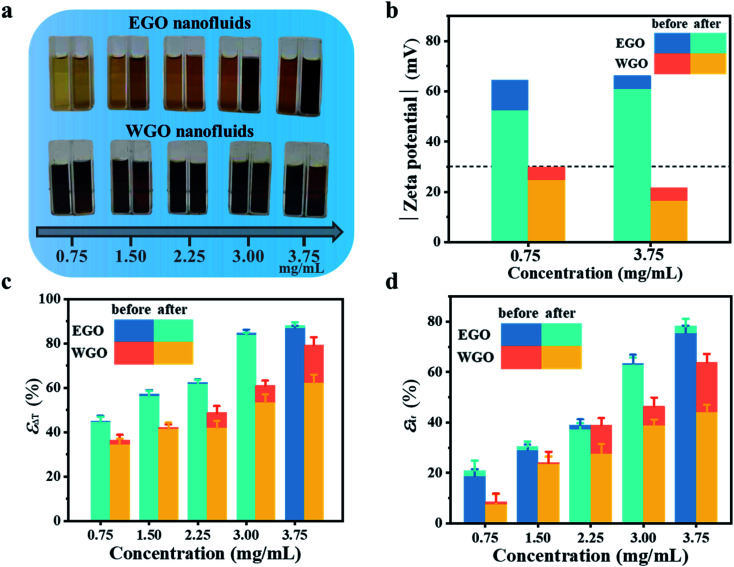
(a) Photographs showing the appearance of EGO and WGO nanofluids before (left) and after (right) repeated freezing/melting for 50 and 30 cycles, respectively. (b) The absolute value of zeta potential for EGO and WGO nanofluids before and after the stability tests (grey dashed line: the required zeta potential of 30 mV for achieving stable dispersion of nanofluids). (c) Comparison of the reduction percentage of supercooling degree for EGO and WGO nanofluids before and after the stability tests. (d) Comparison of the reduction percentage of freezing time for EGO and WGO nanofluids before and after the stability tests.

Long-term dispersion stability of GO sheets in nanofluids is critical for achieving consistent CTES performance for practical applications. The stability tests were carried out by repeating the freezing/melting of nanofluids. As presented in Fig. 3a, the EGO nanofluids with different concentrations demonstrated consistent uniform dispersion without forming any noticeable sedimentation after 50 cycles, but WGO nanosheets aggregated and precipitated out of the base fluid after only 30 cycles. In the meanwhile, the color of EGO nanofluids was darkened after the stability tests, which should be attributed to the partial removal of oxygen-containing groups during continuous freezing/melting processes. Fig. 3b compares the zeta potential of EGO and WGO nanofluids before and after the stability tests. The EGO nanofluids have a higher zeta potential than that of WGO nanofluids with both low and high concentrations. With a concentration of 3.75 mg mL^−1^, the zeta potential of EGO nanofluids decreased from 66.2 mV to 60.9 mV after 50 cycles, which are much higher than the required value of 30 mV for achieving stable dispersion of the nanofluids.^48,49^ By contrast, WGO nanofluids have zeta potentials of 21.6 mV and 16.3 mV before and after the cycling tests, respectively. Compared with the stacked WGO sheets, the monolayer EGO sheet has more exposed edges, which were decorated with abundant oxygen-containing functional groups. These functional groups are ionized and lead to a higher zeta potential.^50^

The dispersion stability has a profound influence on the CTES performance of the nanofluids. It was observed that the temperature evolution profiles of EGO nanofluids with different concentrations were nearly overlapped after repeated freezing/melting for 10, 20, 30, 40, 50 cycles (Fig. S5†). Correspondingly, EGO nanofluids have demonstrated a consistent supercooling degree (Table S3†) and freezing time (Table S4†). Fig. 3b and c present that *ε*_Δ*T*_ and *ε*_tf_ have negligible variation before and after the repeated freezing/melting tests for 50 cycles. By contrast, the temperature curves of WGO nanofluids dramatically deviated from the initial profiles after repeated freezing/melting tests for 30 cycles due to their poor dispersion stability, and the derivation effect became more pronounced with increasing concentrations (Fig. S6†). The aggregated WGO sheets could not provide a sufficient number of nucleation sites for crystallization of the coolant so that the supercooling degree of WGO nanofluids increased after only 10 freezing/melting cycles. The precipitated WGO sheets could not form connected heat conduction networks, which limits heat release during the crystallization process and prolongs the freezing period. At the concentration of 3.75 mg mL^−1^, even two crystallization plateaus were observed (Fig. S6†), which could be attributed to the non-uniform distribution of nucleation sites within the WGO nanofluids. Fig. 3b and c show that at the concentration of 3.75 mg mL^−1^, the *ε*_Δ*T*_ declined from 79.4% to 62.1% and the *ε*_tf_ sharply decreased from 63.8% to 44.1% after only 30 cycles. With gradual precipitation of aggregated WGO sheets, it can be expected that the enhancement of CTES performance for WGO nanofluids will diminish and the nanofluids will suffer from the same large supercooling degree and long freezing time as the base fluids.

### Theoretical analysis

Compared with the base fluid PCMs, the addition of EGO nanosheets provides extra sites for heterogeneous nucleation and growth of PCM crystals. According to classical nucleation theory, the energy barrier (Δ*G*′) for heterogeneous nucleation can be calculated by:^51^

1 Δ*G*′ = (4π*r*^2^*σ*_s_ − 4π*r*^3^Δ*G*_B_/3) × *f*(*θ*)

where *σ*_s_ denotes the surface energy of involved interfaces, Δ*G*_B_ is the volumetric phase-change Gibbs energy and *r* is the critical nucleus radius of a PCM crystal. *f*(*θ*) is a geometrical factor, which depends on the contact angle (*θ*) between the crystal nucleus and GO sheets as shown below:^52–55^

2 *f*(*θ*) = (1 − cos *θ*)^2^(2 + cos *θ*)/4

**Fig. 4:**
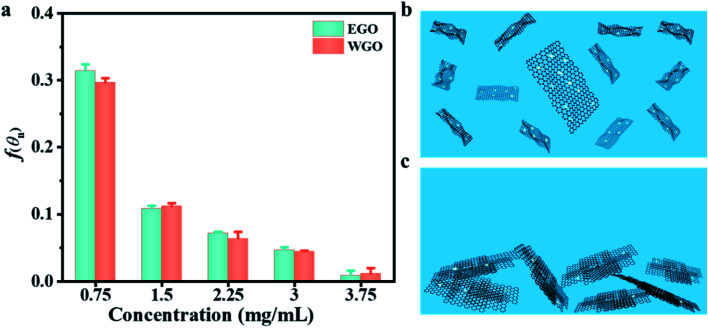
(a) Variation of heterogeneous nucleation-related geometrical factor *f*(*θ*_n_) *versus* concentration for EGO and WGO nanofluids. (b) Schematic showing nucleation of PCM crystals on uniformly dispersed monolayer EGO sheet in nanofluids during the cooling process. (c) Schematic showing nucleation of PCM crystals on aggregated stacked WGO sheets in nanofluids during the cooling process.

A small contact angle can lower the nucleation energy barrier and facilitate the heterogeneous nucleation process. Herein, the contact angle between nanofluids and GO substrates (*θ*_n_), which is slightly larger than *θ*, was measured (Table S5†) and used to evaluate the variation tendency of the geometrical factor.^20^ As shown in Table S5† and Fig. 4a, both the contact angle and the geometrical factor decreased with the increasing concentration of GO sheets, but there is no obvious difference between the EGO and WGO nanofluids at the same concentration.

During the solidification process, the nucleation rate (*J*) of the PCM crystals can be described by:3
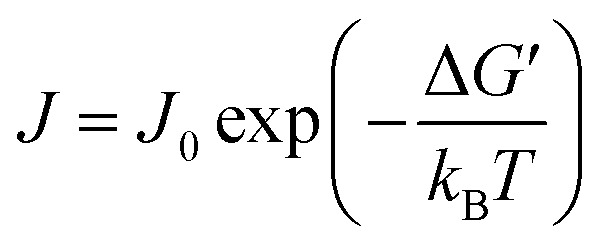
where *k*_B_ is the Boltzmann constant, *T* is the temperature, and *J*_0_ is a kinetic pre-factor that is closely related to the structure and configuration of nucleation sites.^56^ Compared with the stacked WGO sheets, the elastic modulus and bending stiffness of the monolayer EGO sheet are lower^57^ and thus more surface irregularities especially concave cavities can be formed on the flexible EGO surfaces. The PCM nucleation rate in such grooves could be many orders of magnitude larger than that on flat surfaces.^58–60^ This implies that the nucleation rate on each exposed surface of the EGO sheet is much higher than that on the WGO sheet. The distinct dispersion state between EGO and WGO nanofluids further amplifies the disparate nucleation effect. Fig. 4b presents that under the same concentration the exposed surface areas of the monolayer EGO sheet are much larger than that of the stacked WGO sheets (Fig. 4c). The stable uniform dispersion of the monolayer EGO sheet not only enables achieving superior heterogeneous nucleation effect during the solidification process but also helps maintain the consistent thermophysical properties during the repeated freezing/melting process. Both the monolayer structure and the stable dispersion state make EGO nanofluids a potential candidate for high-performance CTES.

## Conclusions

In summary, this work reports stably-dispersed EG nanofluids loaded with low concentrations of monolayer EGO nanosheets as a promising PCM coolant for high-performance subzero CTES. The homogeneous dispersion of monolayer EGO nanosheets simultaneously helps lower the supercooling degree, accelerating the charging/discharging process while retaining the large latent heat of the PCM coolants. The demonstrated stable dispersion overcomes the aggregation and precipitation issue, which is one of the biggest obstacles limiting the practical application of nanofluids technologies. The consistent superior performance, facile preparation process and low loading requirements would make the EGO nanofluids a competitive candidate for high-performance subzero CTES and other important applications.

## Conflicts of interest

There are no conflicts to declare.

## Supplementary Material

RA-011-D1RA04484B-s001
