# Supplementary material for: Ethylene glycol nanofluids dispersed with monolayer graphene oxide nanosheet for high-performance subzero cold thermal energy storage
Source: RSC Adv. 2021 Sep 14;11(49):30495–502. doi: 10.1039/d1ra04484b (PMC9041135; doi:10.1039/d1ra04484b)
Supplement: RA-011-D1RA04484B-s001 [file RA-011-D1RA04484B-s001.pdf]

Electronic Supplementary Material (ESI) for RSC Advances.
This journal is © The Royal Society of Chemistry 2021

Supplementary Information

Ethylene glycol nanofluids dispersed with monolayer graphene oxide nanosheet for high-performance subzero cold thermal energy storage

Jingyi Zhang, Benwei Fu, Chengyi Song, Wen Shang, Peng Tao, Tao Deng**

State Key Laboratory of Metal Matrix Composites, School of Materials Science and Engineering, Shanghai Jiao Tong University, Shanghai, 200240, China

*Email: taopeng@sjtu.edu.cn ; dengtao@sjtu.edu.cn

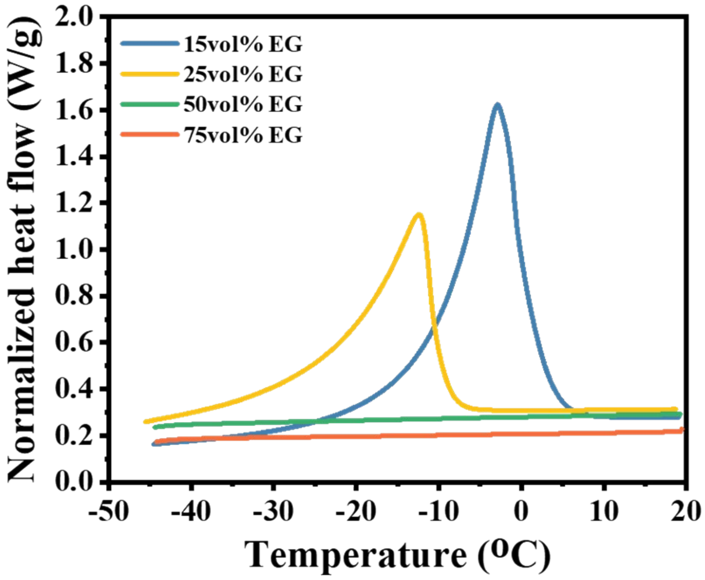

Fig. S1 DSC curves of EG-water solutions with various volume fraction of EG.

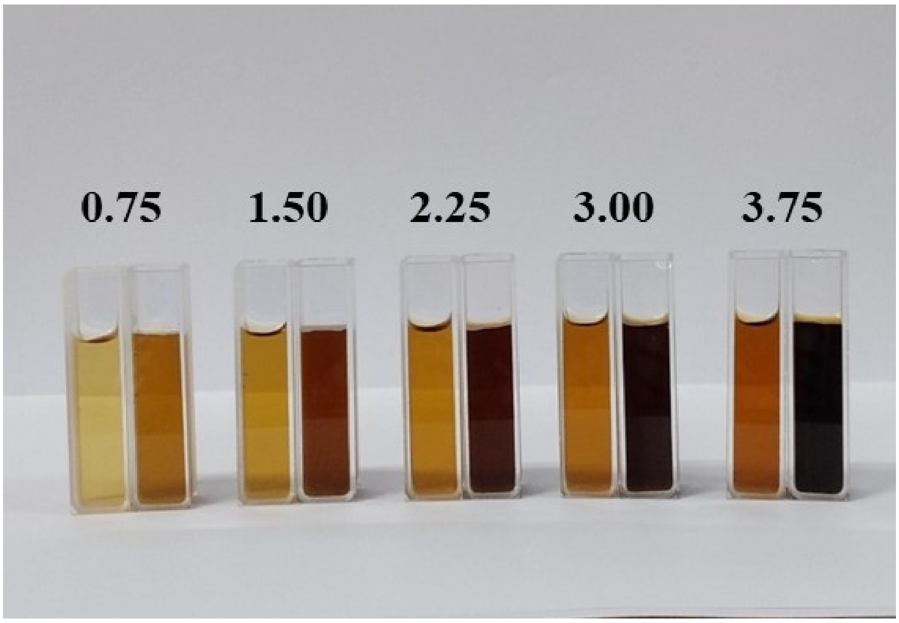

Fig. S2 Photographs of EGO nanofluids (left) and WGO nanofluids (right) with the same concentration. The corresponding concentrations are 0.75 mg/mL, 1.5 mg/mL, 2.25 mg/mL, 3 mg/mL and 3.75 mg/mL, respectively.

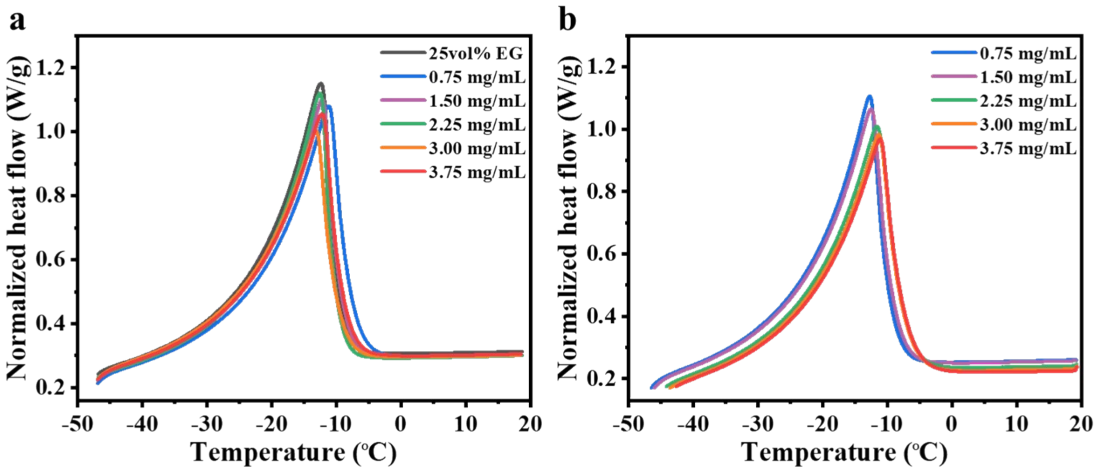

Fig. S3 DSC curves of (a) base coolant solution (25 vol% EG, 75 vol% water) and EGO nanofluids, and (b) WGO nanofluids with various concentration of GO sheets.

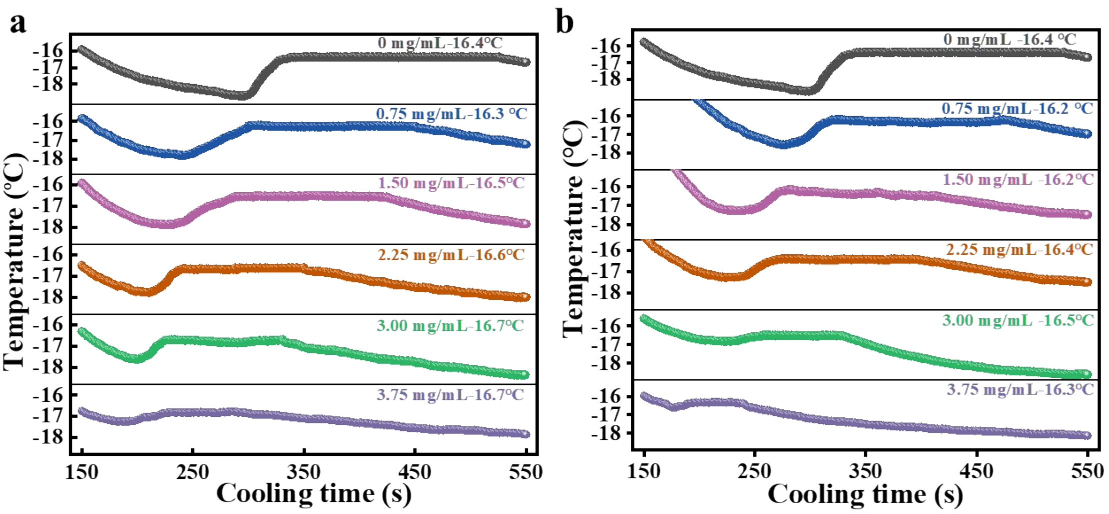

Fig. S4 Temperature evolution profiles of (a) EGO nanofluids and (b) WGO nanofluids during the cooling process. The phase change temperatures for each nanofluid were marked.

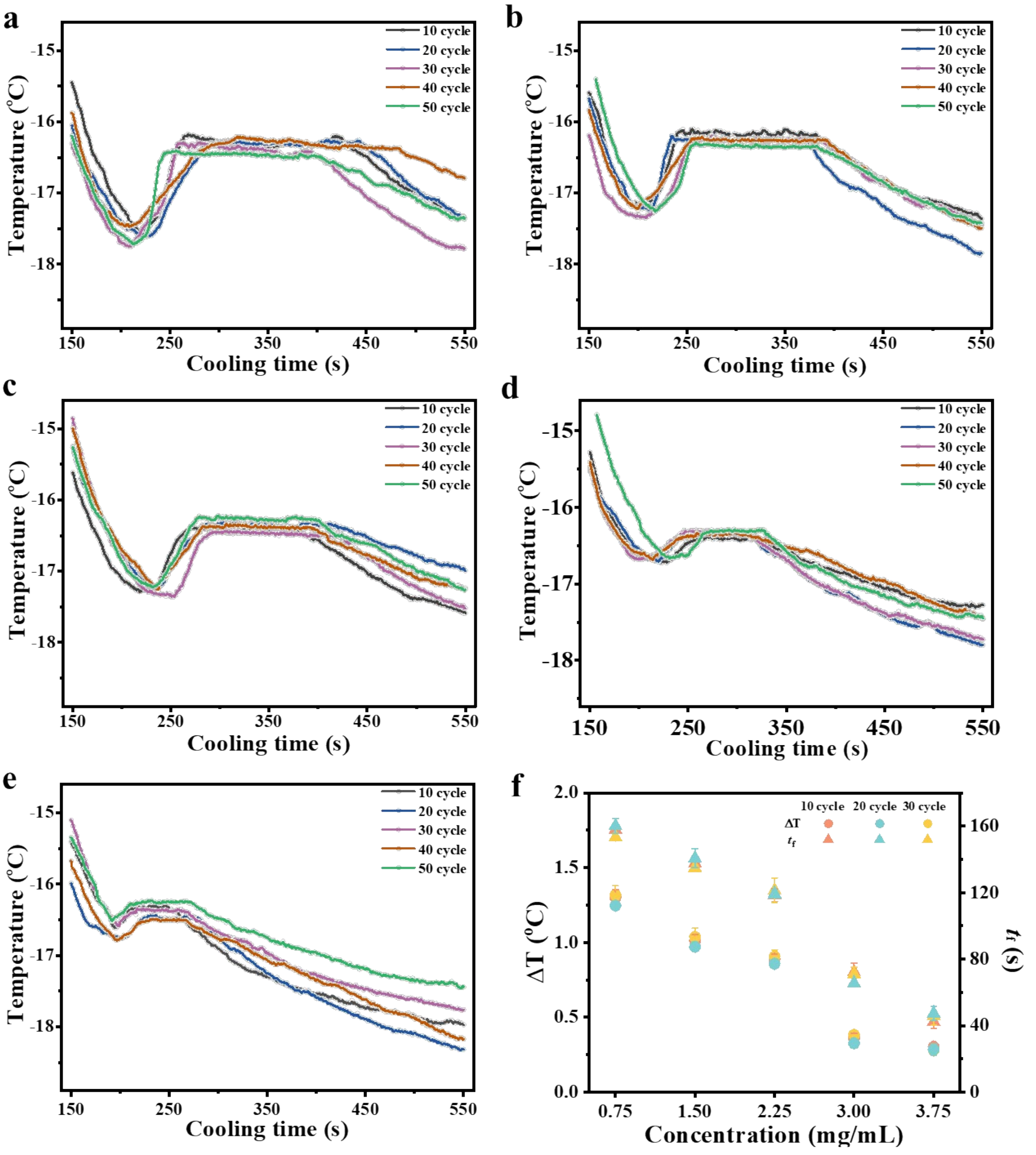

Fig. S5 Temperature evolution profiles of EGO nanofluids with a concentration of (a) 0.75 mg/mL, (b) 1.50 mg/mL, (c) 2.25 mg/mL, (d) 3.00 mg/mL and (e) 3.75 mg/mL during the cooling charging process for different cycles. (f) Comparison of supercooling degree and freezing time of EGO nanofluids before and after stability tests.

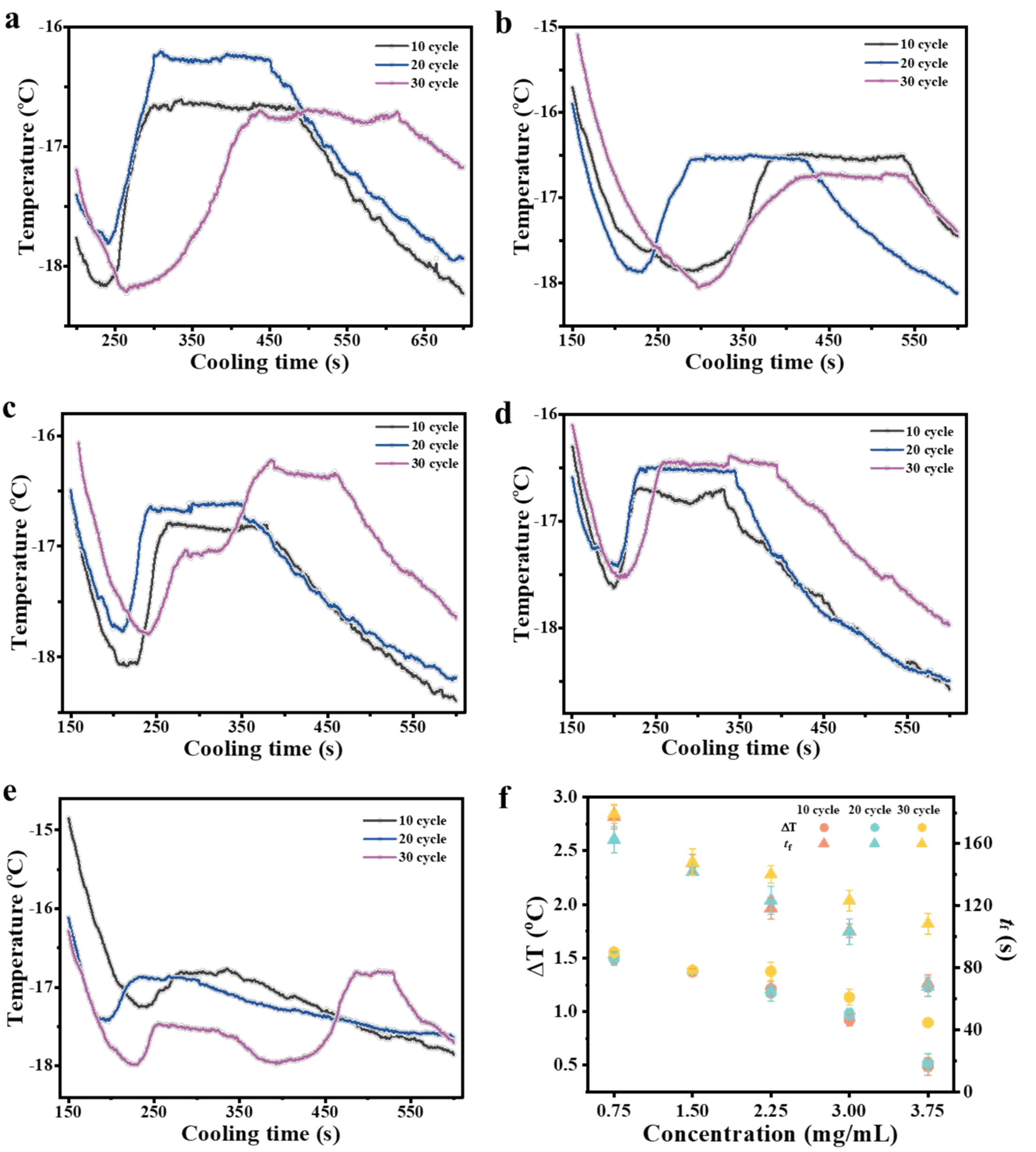

Fig. S6 Temperature evolution profiles of WGO nanofluids with a concentration of (a) 0.75 mg/mL, (b) 1.50 mg/mL, (c) 2.25 mg/mL, (d) 3.00 mg/mL and (e) 3.75 mg/mL during the cooling charging process for different cycles. (f) Comparison of supercooling degree and freezing time of WGO nanofluids before and after stability tests.

Table. S1. Uncertainty analysis of experimental instruments.

Device	Accuracy
Thermostat bath	±0.1 °C
Data acquisition system	±0.01 °C
T-type thermocouple	0.75%

Table. S2 Density and viscosity of EGO and WGO nanofluids

Sample	Density (g/cm³)	Viscosity (mPa·s, 25 °C)
25 vol% EG	1.03	1.544
EGO-EG (0.75 mg/mL)	1.04	1.546
EGO-EG (1.50 mg/mL)	1.04	1.546
EGO-EG (2.25 mg/mL)	1.06	1.547
EGO-EG (3.0 mg/mL)	1.05	1.548
EGO-EG (3.75 mg/mL)	1.06	1.550
WGO-EG (0.75 mg/mL)	1.03	1.548
WGO-EG (1.50 mg/mL)	1.04	1.549
WGO-EG (2.25 mg/mL)	1.04	1.552
WGO-EG (3.0 mg/mL)	1.03	1.554
WGO-EG (3.75 mg/mL)	1.05	1.554

Table. S3 Supercooling degree of EGO and WGO nanofluids after stability tests

			0.75 mg/mL	1.5 mg/mL	2.25 mg/mL	3.0 mg/mL	3.75 mg/mL
ΔT/℃	EGO/ 25 vol% EG	10 cycles	1.30	1.01	0.89	0.36	0.30
		20 cycles	1.31	1.00	0.94	0.40	0.29
		30 cycles	1.32	1.04	0.91	0.38	0.28
		40 cycles	1.17	1.05	0.90	0.38	0.29
		50 cycles	1.25	1.03	0.92	0.38	0.29
	WGO/ 25 vol% EG	10 cycles	1.51	1.37	1.21	0.92	0.49
		20 cycles	1.49	1.38	1.18	0.98	0.53
		30 cycles	1.55	1.39	1.37	1.10	0.90

Table. S4 Freezing time of EGO and WGO nanofluids after stability tests

			0.75 mg/mL	1.5 mg/mL	2.25 mg/mL	3.0 mg/mL	3.75 mg/mL
t_f/s	EGO/ 25 vol% EG	10 cycles	158	138	118	72	42
		20 cycles	160	141	119	66	52
		30 cycles	153	135	121	71	47
		40 cycles	159	138	119	71	48
		50 cycles	158	136	121	72	50
	WGO/ 25 vol% EG	10 cycles	177	147	119	104	70
		20 cycles	163	142	124	103	68
		30 cycles	179	148	140	118	108

Table. S5 Contact angle between nanofluids with different concentrations and graphene oxide sheet substrates

Concentration (mg/mL)	EGO		WGO	
	Contact angle	standard deviation (σ, °)	Contact angle	standard deviation (σ, °)
0.75	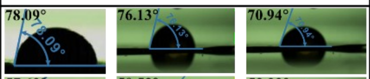	3.02	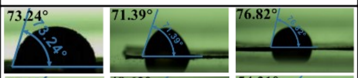	2.25
1.50	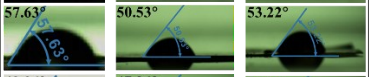	2.93	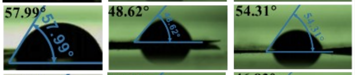	3.85
2.25	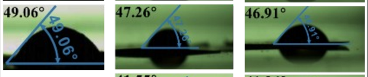	0.94	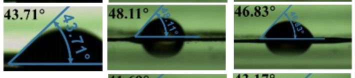	1.85
3.00	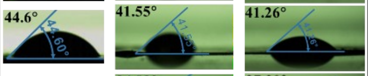	1.51	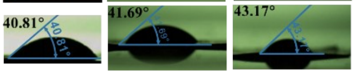	0.97
3.75	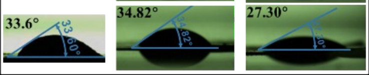	3.30	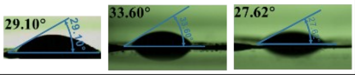	2.54

Note S1

The reduction percentage of supercooling degree ($\varepsilon_{\Delta T}$) was calculated by:

$$\varepsilon_{\Delta T} = \frac{\Delta T_{bf} - \Delta T_{nf}}{\Delta T_{bf}} \times 100\%$$

where ΔT_{bf} and ΔT_{nf} are the supercooling degree of the base fluid and the prepared nanofluids, respectively.

Similarly, the reduction percentage of freezing time (ε_{tf}) was calculated by:

$$\varepsilon_{tf} = \frac{t_{f-bf} - t_{f-nf}}{t_{f-bf}} \times 100\%$$

where $t_{f\text{-}bf}$ and $t_{f\text{-}nf}$ are the supercooling degree of the base fluid and the prepared nanofluids, respectively.
